# Reproductive integration of leptin and kisspeptin in small ruminants: Mechanisms, biomarker potential, and prospects for precision breeding

**DOI:** 10.14202/vetworld.2025.1614-1633

**Published:** 2025-06-19

**Authors:** Herdis Herdis, Ismeth Inounu, Santoso Santoso, Rahma Isartina Anwar, Sari Yanti Hayanti, Mohammad Firdaus Hudaya, Desiana Ade Mahari, Florentina Bety Indah Lupitasari, Anita Hafid, Marchie Asrid da Costa, Nur Adianto, Pradita Iustitia Sitaresmi

**Affiliations:** 1 Research Center for Animal Husbandry, National Research and Innovation Agency, Cibinong Science Center, Jalan Raya Jakarta-Bogor, Bogor, 16915, Indonesia; 2 Department of Reproduction and Obstetrics; Faculty of Veterinary Medicine, Universitas Gadjah Mada, Jalan Fauna No. 2, Karangmalang, Sleman, Yogyakarta, 55281, Indonesia; 3 Department of Food, Agriculture and Fisheries, Ministry of Agriculture, Jalan Letjen S Parman Km. 02, Wonosobo Regency, Jawa Tengah, 56315, Indonesia

**Keywords:** body condition score, estrous regulation, gene polymorphism, gonadotropin-releasing hormone regulation, hypothalamic-pituitary-gonadal axis, kisspeptin, leptin, livestock reproduction, metabolic-reproductive interaction, puberty induction, fertility markers, reproductive biomarkers, selection traits, small ruminants, therapeutic targets

## Abstract

Kisspeptin and leptin (LEP) are two essential proteins that play a central role in regulating reproductive hormones in small ruminants through the hypothalamic-pituitary-gonadal axis. These proteins influence the secretion of gonadotropin-releasing hormone, which, in turn, controls key hormones such as follicle-stimulating hormone and luteinizing hormone. Acting in synergy, kisspeptin and LEP also interact with other metabolic and reproductive signals, including insulin, estrogen, and neuropeptides, to coordinate reproductive function. Despite their importance, the detailed mechanisms by which these proteins operate, especially in relation to body condition score are not yet fully understood. This review explores their biological roles, interactions, and potential as markers for selecting high-performing livestock. External factors such as diet, stress, and seasonal changes can further influence their expression and activity. Understanding these pathways can support improved fertility management and the development of genetic or therapeutic strategies to enhance reproductive efficiency in goats and sheep.

## INTRODUCTION

The selection of livestock based on reproductive parameters is essential for the efficient utilization of time and resources [1]. Specific genetic traits and protein markers significantly influence reproductive efficiency, particularly in small ruminants. Gonadotropin-releasing hormone (GnRH) is a key protein that regulates the secretion of gonadotropin hormones and is vital to the reproductive cycles of these animals. The secretion of gonadotropins – such as luteinizing hormone (LH), follicle-stimulating hormone (FSH), and inhibin – is primarily governed by GnRH [2]. In addition to GnRH, other hypothalamic regulators such as gonadotropin-inhibiting hormone (GnIH) and kisspeptin have been identified; GnIH suppresses, while kisspeptin enhances, GnRH secretion – underscoring kisspeptin’s pivotal role in reproductive regulation. Leptin (LEP) is another essential protein known to influence GnRH activity through its receptor (LEP receptor [LEPR]) and is closely associated with key reproductive traits, including lactation performance, calving frequency, and age at first parturition. Given the physiological importance of kisspeptin and LEP in the hypothalamic-pituitary-gonadal (HPG) axis, their roles in small ruminant reproductive function merit further investigation [3].

Although the roles of kisspeptin and LEP in reproductive regulation have been well-documented in various mammalian species, there remains a lack of comprehensive synthesis focusing specifically on small ruminants. The dynamic interactions between these two proteins and their collective influence on GnRH, LH, and FSH secretion under varying physiological and environmental conditions, such as body condition score (BCS), nutritional status, and photoperiod, have not been thoroughly explored. Furthermore, the genetic polymorphisms and tissue-specific expression patterns of kisspeptin (*KiSS-1*) and *LEP* genes, particularly their implications for fertility traits, remain underreported in the context of goats and sheep. This represents a significant gap in the literature on reproductive physiology and animal genetics.

**Figure 1:**
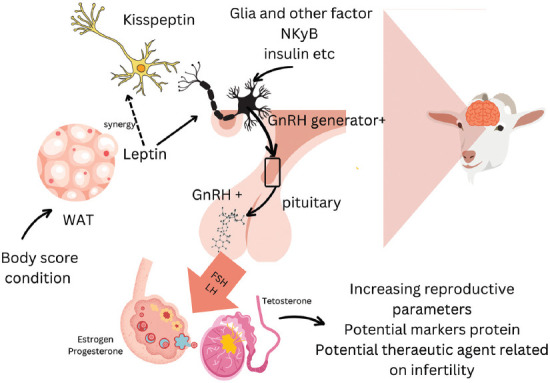
Graphical abstract.

This review aims to critically synthesize current knowledge on the roles of kisspeptin and LEP in the reproductive physiology of small ruminants. Specifically, it examines their regulatory mechanisms within the HPG axis, their interactions with metabolic cues and BCS, as well as their genetic and molecular characteristics. In addition, the review highlights their potential utility as biomarkers and therapeutic targets for improving reproductive efficiency and guiding precision breeding strategies in small ruminants. The graphical abstract is presented as Figure 1.

## KISSPEPTIN: ORIGIN, BIOLOGICAL ROLES, AND FUNCTION IN SMALL RUMINANTS

Kisspeptin, also known as metastin, was originally identified as an anti-metastatic peptide by Lee *et al*. in 1996 [4]. It was later recognized as a critical neuroendocrine regulator of GnRH signaling, particularly in stimulating the secretion of LH. In small ruminants, kisspeptin plays a central role in reproductive regulation by acting on hypothalamic neurons. The peptide is encoded by the *KiSS-1* gene, a member of the RF-amide peptide family, and acts through its receptor G protein-coupled receptor 54 (GPR54), which is highly expressed in the hypothalamus and actively participates in the HPG axis [5]. In humans, the *KiSS-1* gene is located on chromosome 1q32 [6], whereas in goats and sheep, it is mapped to chromosome 16 [7].

Kisspeptin neurons are primarily located in the hypothalamus, although some studies have reported limited expression in placental tissues. Wakabayashi *et al*. [8] localized kisspeptin neurons predominantly to the arcuate nucleus (ARC) in goats. Complementary findings by Ohkura *et al*. [9, 10] confirmed the distribution of kisspeptin-expressing neurons in both the ARC and preoptic area (POA) of male goats. Expression of the *KiSS-1* gene has also been detected in the POA of female goats, although this remains a topic of scientific debate. These neurons are primarily concentrated in the caudal ARC, dorsomedial nucleus, and medial POA (mPOA) [11]. In sheep, kisspeptin-expressing cells are predominantly found in the periventricular nucleus (POV), in close proximity to GnRH neurons. Notably, in goats, the expression of *KiSS-1* or its product KP-54, through the GPR54 receptor, is higher in the hypothalamus than in peripheral reproductive tissues such as the ovary, oviduct, and endometrium [12].

The influence of *KiSS-1* on GnRH secretion has been demonstrated in both goats [13] and sheep [14]. Although polymorphisms in exon 1 of the *KiSS-1* gene in goats have been investigated since 2009, their functional implications remain unclear [15]. Several mutations have been identified: three in intron 1 and two in exon 3. While some are considered non-causal, they may still affect nearby regulatory elements that influence reproductive function [16]. Experimental study by Han *et al*. [13] indicates that administration of kisspeptin at concentrations ranging from 1 μM to 100 μM enhances the steroidogenic activity of Leydig cells in goat testes, where *KiSS-1* gene expression has also been confirmed [17]. Although kisspeptin activity has been detected in Sertoli cells, spermatids, and spermatozoa in other species, its presence in these cells in goats remains unverified [18]. Collectively, these findings suggest that the *KiSS-1*/GPR54 system may exert both central and peripheral regulatory effects on reproduction.

In ewes, administration of kisspeptin analogues such as TAK-683 and C6 has been shown to induce estrus, indicating potential reproductive applications in goats. Moreover, ovariectomized ewes exhibited elevated LH and GnRH levels in cerebrospinal fluid following intravenous injection of KP-10, reinforcing kisspeptin’s stimulatory role in reproductive hormone release [19].

## LEP: ORIGIN, MOLECULAR BASIS, AND REPRODUCTIVE FUNCTION IN SMALL RUMINANTS

LEP is a protein hormone encoded by the *LEP* gene, located on chromosome 7q31.3. It was initially identified in human adipose tissue in 1994 and later in goats in 2005, where it was characterized as a 16-kDa protein [12, 20]. In Raini cashmere goats, *LEP* gene expression has been observed across multiple tissues, including adipose tissue, liver, kidney, lung, and heart, with the highest expression detected in adipose tissue and liver, and the lowest in the heart [21]. LEP is synthesized as a prohormone comprising 167 amino acids, which is processed into its biologically active form consisting of 146 amino acids [22].

In the context of reproduction, LEP acts as a neuromodulator by binding to its receptors and influencing neuropeptide Y (NPY), a critical regulator of gonadal activity in the central nervous system [23]. Its primary physiological role is to convey the animal’s nutritional status to the brain, thereby modulating key reproductive functions such as the onset of puberty and the secretion of gonadotropins, particularly LH and FSH. Polymorphisms within intron 1 of the *LEP* gene have been associated with metabolic and endocrine traits, including variations in beta-hydroxybutyrate (BHBA), free thyroxine (fT4), insulin-like growth factor-1 (IGF-1), triglyceride levels, and milk somatic cell counts [24]. Additionally, the T117C polymorphism has been associated with differences in milk yield performance at 140 days in goats [25].

LEPRs in the ARC of the hypothalamus interact with NPY and glucagon-like peptide-1, integrating energy balance and reproductive signals. Under growth hormone (GH) activation, LEP also functions as a negative regulator of GnRH secretion and has been reported to suppress cortisol production [26]. The connection between LEP and reproduction was first recognized through studies on placental LEP secretion, which demonstrated LEP resistance during mid-gestation in cattle. This resistance results in increased appetite despite weight gain, reflecting the elevated nutritional demands of pregnancy [27].

Moreover, LEP directly stimulates GnRH receptor expression and FSH secretion by inhibiting the translation of Musashi protein mRNA in goats. In females, elevated LEP concentrations promote estrogen production, thereby inducing the LH surge necessary for ovulation. LEP also plays an indirect role in oocyte maturation. In both sexes, LEP is a critical regulator of puberty onset. Experimental reductions in LEP levels by 50%–90% have been shown to delay sexual maturation, suppress gonadal activity, and hinder testicular and ovarian development. However, excessive LEP levels, such as those observed in obesity, can lead to LEP resistance, which may impair reproductive function [28].

## EXPRESSION AND LOCALIZATION OF KISSPEPTIN AND LEPRS IN SMALL RUMINANTS

Research in goats has predominantly focused on two major *KiSS-1* gene products: KP-10 and KP-54 [29]. KP-10 is a decapeptide with a molecular weight of 1302.4 g/mol, whereas KP-54 consists of 54 amino acids and weighs approximately 5857 g/mol [30]. Administration of KP-10 during the luteal phase has been shown to stimulate the secretion of LH and FSH, without significantly altering levels of GH or prolactin [31]. Another variant, KP-135, contains 135 amino acids and has a molecular mass of approximately 14.38 kDa [32].

**Figure 2:**
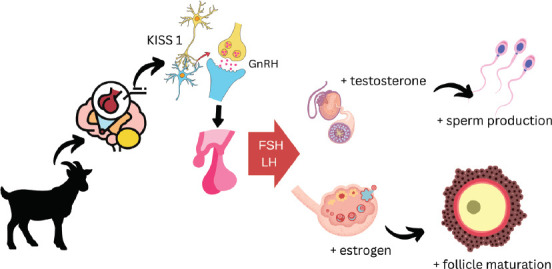
Kisspeptin mechanism in goat reproduction [Source: The figure was generated by the authors].

Comparative sequence analysis indicates that the goat *KiSS-1* nucleotide and amino acid sequences share greater similarity with those of other livestock species than with those of humans or rodents. The goat *KiSS-1* polypeptide includes a signal peptide, supporting its classification as a precursor for secreted bioactive peptides [18]. Structurally, goat kisspeptin is predicted to contain an LRY-amide motif at its C-terminal end, a feature conserved across rodent and bovine species. The full-length *KiSS-1* gene transcript in goats spans approximately 408 base pairs, comprising two coding exons and one intron, encoding a 135-amino acid peptide [7]. The mechanism by which kisspeptin regulates reproduction in goats is illustrated in Figure 2.

LEP, primarily produced and secreted by adipocytes, plays a critical role in energy homeostasis and reproductive function [24]. Its expression has been extensively documented in various livestock species, including small ruminants, where genotypic diversity and metabolic adaptation have been noted [33]. In these species, the *LEP* gene is located on chromosome 4 and comprises three exons [34]. Fluorescence *in situ* hybridization has localized this gene specifically to chromosome 4q32. In Bligon goats, four novel single-nucleotide polymorphisms (SNPs) have been identified within introns 1 and 2 of the *LEP* gene [35]. These intronic polymorphisms are associated with important metabolic traits, including BHBA, fT4, IGF-1, triglyceride concentrations, and milk somatic cell counts [36].

**Figure 3:**
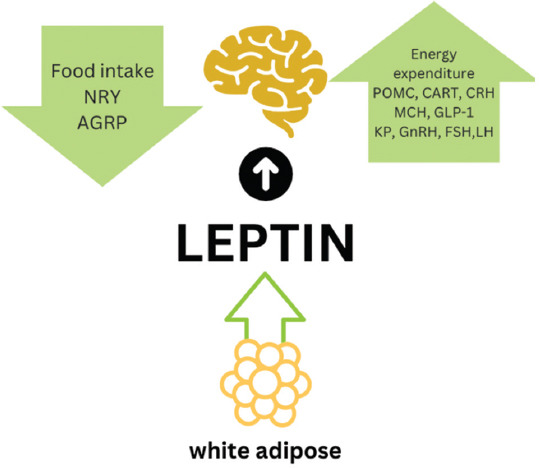
Mechanism of leptin in small ruminant bodies [Source: The figure was generated by the authors].

Plasma LEP concentrations serve as permissive signals for the initiation of sexual maturity in small ruminants [28], particularly during gestation and lactation, when LEP levels are significantly elevated between days 2 and 9 postpartum [24]. LEP also facilitates the attainment of a critical body size threshold necessary for the onset of puberty [28]. Notably, livestock subjected to restricted nutrition may still achieve puberty on exogenous LEP administration. This response involves cross-talk between LEP signaling and estrogen receptors in the gonads and hypothalamus, as well as circulating IGF-1 levels [37]. Administration of physiological doses of LEP (0.2–2 ng/mL) has been reported to increase LH secretion by approximately 1.5-fold, whereas higher doses exceeding 20 ng/mL produce no significant reproductive effects [38]. The detailed mechanism by which LEP regulates reproductive pathways in small ruminants is depicted in Figure 3.

## RECEPTOR EXPRESSION AND LOCALIZATION

The receptor for kisspeptin, GPR54 – also referred to as kisspeptin receptor (KiSS-1r) – exhibits notable genetic polymorphisms in female goats, including five mutations within exon 1 and a partial mutation in exon 5 [39]. GPR54 encodes a transmembrane G-protein-coupled receptor and is expressed more abundantly in the ovaries compared to the hypophysis, oviduct, and endometrium of female goats [12]. Both *KiSS-1* and *GPR54* genes are expressed across several brain regions, with prominent localization in the hypothalamus, basal ganglia, and arcuate nuclei. In the hypophysis, GPR54 is predominantly found in the median eminence (ME), particularly within GnRH neurons [40], while peripheral expression has also been reported outside the cerebral vasculature [41]. In ewes, expression of *KiSS-1* and *GPR54* has been documented in the pituitary gland, endometrium, ovaries, and oviducts [12, 29]. High expression levels of GPR54 have been observed in the brain and in several peripheral tissues as well [42]. An immunolocalization study by Han *et al*. [13] further confirms the presence of kisspeptin in Leydig and Sertoli cells, as well as in spermatids of goats.

Kisspeptin fibers are secreted into the portal vasculature, with dense clusters of immunoreactive fibers found outside the ME [43]. In various species, kisspeptin-secreting neurons are located in the internal zone of the hypothalamus, allowing for interregional neuroendocrine communication. These fibers are especially concentrated in the ARC, proximal to kisspeptin neuronal somata. Most GnRH neurons are located in the septo-POA, where kisspeptin fibers are also frequently detected [44]. In addition, a major population of kisspeptin fibers runs adjacent to the third ventricle, and all fibers within the ME appear to originate from the ARC [45], indicating minimal contribution from other hypothalamic regions. Anterograde tracing in ewes further supports direct projections from ARC kisspeptin neurons to the ME [45]. Moreover, kisspeptin/neurokinin B/dynorphin (KNDy) neurons have been identified as the primary afferent inputs to preoptic kisspeptin neurons [46, 47].

The long-form LEPRb, a member of the class I cytokine receptor family, exists in six isoforms and is the functionally active variant in reproduction [48]. In Black Bengal goats, two polymorphisms in intron 3 and one in exon 4 of the *LEPRb* gene have been associated with variation in litter size [49]. LEPRb mRNA has been detected in reproductive tissues such as the ovaries and mammary glands of female goats [25, 50]. Within the hypothalamus, LEPRs are highly expressed and play a central role in modulating GnRH secretion [28]. LEPRb is localized in the ME-ARC and shows heightened responsiveness in animals with adequate nutritional status. Under conditions of fasting or negative energy balance, LEP signaling supports the recovery of hypothalamic function and GnRH pulsatility, which may otherwise be suppressed through GABAergic pathways [51]. Additional LEP-mediated modulation of GnRH secretion is facilitated by neuropeptides such as melanocyte-stimulating hormone, cocaine- and amphetamine-regulated transcript, and galanin-like peptide [52].

In small ruminants, LEPRs are primarily localized to the gonadotroph cells in the pars tuberalis of the adenohypophysis (70%–90%), and to a lesser extent in the pars distalis (<30%). In the ovaries, LEPRs are found in corpus luteum cells and are involved in promoting oocyte maturation. Moreover, LEP supports the structural development and functional maintenance of the corpus luteum [37, 53]. Expression of LEPRs has also been confirmed in granulosa and theca cells, oocytes, and placental tissues, underscoring its broad regulatory influence on reproductive physiology.

## SYNERGISTIC INTERACTIONS IN REPRODUCTIVE REGULATION

Kisspeptin and LEP act synergistically to stimulate the HPG axis, thereby promoting the secretion of key reproductive hormones. This interaction underscores the coordinated regulatory role of both proteins in governing reproductive functions in small ruminants [54]. LEP, secreted predominantly by adipose tissue, indirectly influences GnRH-secreting neurons in the hypothalamus, even though LEP itself is not expressed in these neurons. Instead, LEP’s effects are mediated through neural circuits that intersect with the kisspeptin signaling pathway through the KiSS-1/GPR54 axis.

This metabolic-reproductive interface highlights how body condition and nutritional status modulate reproductive function. Under normal physiological conditions, LEP downregulates *NPY* expression, thereby reducing appetite and enhancing reproductive activity. Evidence suggests a direct association between LEP protein levels and kisspeptin receptor activity. Notably, intracerebral LEP administration in undernourished sheep has been shown to upregulate *KiSS-1* gene expression [55].

Experimental studies have demonstrated that administering LEP at concentrations between 10^-8^ M and 10^-7^ M significantly increases kisspeptin secretion, with levels peaking at approximately 30 h – rising from 36 to over 175 pg. However, concentrations outside this range (e.g., 10^-5^ M) reduce kisspeptin secretion to levels below baseline, indicating a dose-dependent, biphasic relationship between LEP and kisspeptin [54]. These findings emphasize the importance of maintaining an optimal BCS to ensure efficient reproductive function, as balanced LEP levels are essential for activating kisspeptin-mediated pathways.

Comparable studies by Backholer *et al*. [56] and Scott *et al*. [57] have shown that intracerebroventricular LEP infusion in lean ewes results in increased kisspeptin expression, although at lower levels than in adequately nourished animals, and subsequently enhances LH secretion. The interaction between LEP and kisspeptin can thus be summarized as follows: nutritional status influences LEP levels, which interact with the LEPR to modulate kisspeptin signaling. This, in turn, regulates GnRH secretion and downstream release of FSH and LH, ultimately affecting estrogen and testosterone production [58].

**Figure 4:**
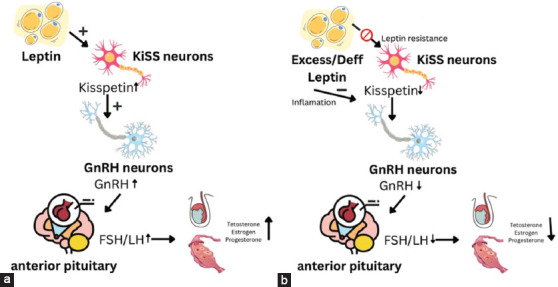
Mechanism of kisspeptin and leptin synergy. (a) Normal physiology and (b) obese or lean pathology in small ruminants [Source: The figure was generated by the authors].

Interestingly, this synergistic relationship appears to be functionally active only after puberty. During early developmental stages, LEP signaling and receptor expression are limited, despite the presence of kisspeptin receptors in the hypothalamus. Cravo *et al*. [59] suggest that LEP may contribute to pubertal onset by promoting STAT3 phosphorylation, which is involved in upregulating *KiSS-1* receptor expression, thereby initiating sexual maturation. Nonetheless, this mechanism remains a subject of ongoing investigation. The integrated signaling between kisspeptin and LEP is illustrated in Figure 4.

## MECHANISMS OF KISSPEPTIN AND LEP IN SMALL RUMINANTS

Kisspeptin-producing (*KiSS-1*) neurons in the forebrain serve as critical intermediaries in regulating the HPG axis, responding to diverse internal and external cues, such as gonadal steroid hormones and photoperiodic changes. In males, kisspeptin facilitates testicular development and testosterone synthesis through the HPG axis, paralleling its function in female reproductive regulation [13]. In small ruminants, kisspeptin is highly expressed in Leydig cells, and its inhibition significantly reduces testosterone secretion [17]. Similar effects have been observed in the ovarian tissues of females, indicating a conserved regulatory mechanism across sexes [60]. Within the hypothalamus, kisspeptin neurons are primarily localized in the POA and ARC, where they co-express estrogen receptor α, progesterone, and androgen receptors. These neurons govern GnRH neuron activity, thus regulating the broader endocrine cascade of the HPG axis [61].

Kisspeptin mediates feedback regulation from steroid hormones and stimulates FSH secretion; however, it exhibits a stronger and more immediate effect on LH release. Central administration of kisspeptin results in delayed FSH secretion relative to LH, and *in vivo* data suggest that FSH is approximately 200-fold less sensitive to kisspeptin stimulation compared to LH [62, 63]. The mechanisms underlying this disparity may include intrinsic differences in LH and FSH release patterns, kisspeptin’s preferential promotion of high-frequency GnRH pulses, which favor LH, and the modulatory influence of gonadal peptides, such as inhibins, on FSH secretion. In addition to its central expression, kisspeptin is present in extra-hypothalamic sites, including the placenta, specifically in syncytiotrophoblasts and trophoblast giant cells, and the pancreas, where its receptor GPR54 is also expressed [64].

Emerging evidence supports the role of ARC kisspeptin neurons as part of the GnRH pulse generator. This is substantiated by multi-unit activity recordings and rhythmic intracellular Ca^²+^ oscillations in ARC neurons corresponding to LH pulses in goats [65, 66]. Optogenetic activation and inhibition of these neurons have been shown to initiate or suppress pulsatile LH secretion, respectively. Functional diversity among kisspeptin isoforms, such as KP-10, KP-13/16, and KP-53/54, adds further complexity, although the mechanisms behind these differences remain incompletely understood. Species-specific variation in kisspeptin amino acid sequences, particularly in the C-terminal pharmacophore region of KP-10 in sheep and goats, suggests differential biological activity that may necessitate tailored administration strategies [67].

LEP, although exerting its reproductive effects primarily through hypothalamic action, is not directly expressed in GnRH neurons. This indicates that intermediary pathways, particularly involving kisspeptin, are responsible for transmitting LEP’s signals to the reproductive axis [68]. Kisspeptin may thus serve as a key link between LEP signaling and reproductive hormone regulation. This model aligns with findings implicating the LEP/LEPR and kisspeptin/KiSS-1 receptor systems as central to puberty initiation. The pharmacophore region of kisspeptin, which binds to and activates GPR54, initiates intracellular cascades that drive GnRH secretion—an essential process for the onset of puberty and reproductive maintenance [69].

Kisspeptin also acts as a metabolic sensor, convey-ing information about energy status to the central nervous system. It regulates positive and negative feedback loops of gonadal steroids and is sensitive to nutritional changes. Under physical stress or energy deficits (e.g., excessive exercise and fasting), *KiSS-1* expression may decrease, impairing fertility [70, 71]. Pubertal initiation and activation of the HPG axis are closely linked to body fat and energy balance. Elevated serum kisspeptin levels have been observed in obese individuals, indicating an overabundance of energy reserves [72]. In obese mice, increased LEP and inflammatory cytokines reflect an excess energy status; however, paradoxically, obesity may suppress kisspeptin expression and impair HPG axis function due to LEP resistance and inflammatory interference [73].

Both short- and long-term changes in energy balance modulate LEP signaling, which feeds back to the hypothalamus to influence reproductive hormones. In LEP-deficient obese mice, reduced kisspeptin expression and neuron count have been observed [73]. Although kisspeptin neurons appear to contain LEPRs, their function is compromised in states of LEP resistance, affecting both central and peripheral systems [74]. LEP replacement therapy in such models has restored fertility by correcting GnRH secretion defects, underscoring the pivotal role of intact LEP–kisspeptin signaling in reproductive competency [75].

### BCS and hormonal regulation in small ruminants

BCS is a practical and widely adopted tool for evaluating the nutritional and physiological status of small ruminants, such as goats and sheep [76]. BCS significantly influences reproductive performance by modulating the secretion of key metabolic and reproductive hormones, particularly LEP and kisspeptin. LEP, secreted primarily by adipose tissue, serves as a signal of energy reserves to the brain, while kisspeptin, a hypothalamic neuropeptide, plays a central role in stimulating GnRH secretion, which subsequently drives the release of FSH and LH.

### Correlation between BCS and LEP in small ruminants

LEP is a critical protein hormone involved in regulating appetite, energy balance, and body weight in both humans and livestock, including small ruminants. Acting through central pathways in the hypothalamus, LEP signals satiety and reduces food intake, thereby directly impacting BCS. It is primarily produced by white adipose tissue, although its gene expression has also been documented in embryonic tissues [78], mammary glands [79], the intestine, abomasum, duodenum, and hypothalamus in ruminants [80]. LEP levels reflect the animal’s nutritional and health status, making it a valuable indicator in livestock management for optimizing diet, body mass, and reproductive efficiency [77].

A robust positive correlation between serum LEP concentrations and fat mass has been established across various species, including humans [81], rats [82], sheep [83], and cattle [84]. Beyond its metabolic role, LEP modulates a wide range of physiological processes, such as endocrine regulation, immune responses, reproductive function, renal activity, hematopoiesis, and angiogenesis [85]. LEP exerts anorexigenic effects and enhances sympathetic nervous system activity, thereby increasing basal metabolism and energy expenditure. These energy-related signals are integrated within the hypothalamic network to activate neuroendocrine pathways controlling reproductive function [86].

Gallego-Calvo *et al*. [87] reported that animals with reduced BCS exhibited impaired ovarian responses, lower estrus expression, and diminished reproductive performance. However, the lack of variation in plasma LEP among experimental groups suggests that other factors may influence these reproductive parameters. Consistent with this, a prior study by Henry *et al*. [88] indicate that short-term energy restriction, such as fasting for 32 h or reduced feed intake over 94 days [89], does not significantly alter plasma LEP levels in lean sheep.

Nevertheless, BCS remains a reliable indicator of energy balance and stress adaptation. Gámez-Vázquez *et al*. [90] demonstrated a direct correlation between BCS and plasma LEP concentrations in goats. LEP plays a key role in pubertal onset, and its serum levels are positively correlated with BCS during the breeding season. This relationship has been further validated by Zhang *et al*. [91] and Towhidi *et al*. [92] in sheep, who found significant correlations between BCS, LEP, and FSH levels in Iranian fat-tailed ewes during mating.

### Correlation between BCS and kisspeptin in small ruminants

Current research highlights the pivotal role of kisspeptin as an intermediary regulator of GnRH secretion in the hypothalamic control. Nutritional deficits, such as those induced by food restriction or prolonged fasting, lead to a decline in BCS, which subsequently suppresses kisspeptin gene expression and peptide production [47]. This inhibition disrupts the GnRH –LH axis, particularly during the anestrus season, resulting in reduced frequency and amplitude of GnRH and LH pulses. The hypothalamus integrates multiple metabolic signals, including circulating insulin and LEP levels, to assess the animal’s nutritional status and modulate GnRH release through kisspeptin signaling. In ewes, dietary restrictions have been shown to impair hormone secretion and suppress gonadotropin release [93].

BCS is closely linked to reproductive performance in small ruminants. Animals with low BCS often experience delayed puberty, reduced conception rates, and irregular estrous cycles. Conversely, individuals with optimal BCS display enhanced reproductive efficiency and consistent cyclicity. Kisspeptin plays a central role in initiating this reproductive activity by stimulating GnRH secretion, which drives the subsequent release of LH through the hypophysial portal circulation. In sheep and goats, kisspeptin neurons are primarily distributed in the mPOA and ARC [94].

Expression of the *KiSS-1* gene and its encoded peptide is seasonally regulated. During the anestrus period, kisspeptin expression in the ARC is significantly reduced compared to the breeding season. Experimen-tal administration of kisspeptin in anestrus females has been shown to induce ovulation, suggesting that seasonal fluctuations in receptor expression and peptide availability modulate responsiveness. The heightened effect of kisspeptin during the non-breeding season may be attributed to the increased expression of its receptor, GPR54, in GnRH neurons. Supporting this, a previous study by Smith *et al*. [95] reported a 5-fold increase in the number of kisspeptin-expressing neurons in the ARC during the breeding season compared to the non-breeding season. The limited number of kisspeptin neurons during anestrus may contribute to variability in detection and quantification across studies.

In addition to its reproductive function, kisspeptin and GPR54 also influence seasonal and metabolic regulation. GPR54 is not only highly expressed in reproductive tissues but also detected in the pancreas, where kisspeptin potentiates glucose-stimulated insulin secretion, implicating a role in pancreatic β-cell activity [96]. These findings emphasize the responsiveness of kisspeptin expression to nutritional cues. Consequently, fluctuations in BCS can alter kisspeptin signaling pathways, potentially impacting reproductive performance and metabolic homeostasis in small ruminants.

## RELATIONSHIP BETWEEN KISSPEPTIN, LEP, AND BCS IN SMALL RUMINANT REPRODUCTION

### Kisspeptin actions in small ruminant reproduction

**Figure 5:**
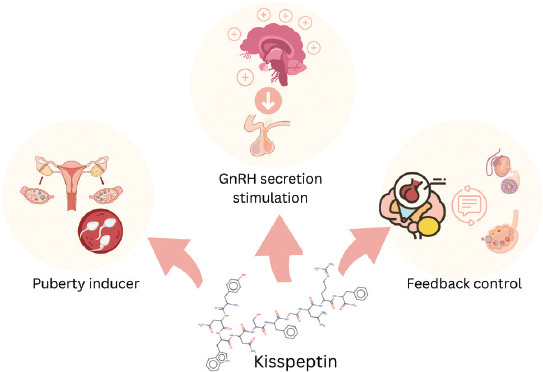
Kisspeptin actions during small animal reproduction [Source: The figure was generated by the authors].

Kisspeptin was initially identified for its role in inhibiting tumor metastasis. Subsequent discoveries have established its critical involvement in reproductive physiology, particularly through its stimulation of GnRH and LH secretion. In small ruminants, LH is released from the anterior pituitary as part of the neuroendocrine cascade that governs reproductive processes (Figure 5). Increasing attention has been paid to the role of kisspeptin in the metabolic regulation of reproduction, puberty onset, and sex steroid feedback modulation. Kisspeptin and its receptor GPR54 have emerged as essential components in the control of the reproductive axis in ruminants.

### Kisspeptin as a puberty inducer

During the onset of puberty, kisspeptin-expressing neurons in the anteroventral periventricular nucleus (AVPV) of both male and female animals reach adult-like levels, indicating their involvement in stimulating GnRH neurons, which are critical for pubertal initiation. In ruminants, the pubertal transition is marked by a reduced sensitivity to estradiol’s inhibitory feedback on LH secretion. Kisspeptin, along with neurokinin B (NKB), which is co-expressed in many hypothalamic neurons, appears to facilitate this transition [97]. Hu *et al*. [98] have confirmed that kisspeptin induces puberty by activating GnRH release, which in turn stimulates pulsatile secretion of LH and FSH through GPR54 signaling.

In male knockout mice lacking *KiSS-1* or *GPR54*, testicular development and testosterone production are arrested, further supporting the central role of kisspeptin in pubertal regulation [13]. While some studies by Samir *et al*. [17], Greives *et al*. [99], and Ando *et al*. [100] suggest minimal GPR54 expression in Leydig cells, this remains a topic of scientific debate [101]. Nonetheless, strong kisspeptin activity has been observed in round spermatids within the seminiferous tubules, and GPR54 expression has been confirmed in Sertoli cells, implying a possible role in enhancing sperm maturation and motility [13, 102]. Furthermore, elevated levels of kisspeptin and GPR54 have been detected in adult goats compared to prepubertal counterparts, coinciding with higher testosterone concentrations, supporting the hypothesis that kisspeptin contributes to pubertal initiation [18]. In a previous study, administration of 10 μM kisspeptin for 24 h increased testosterone production and upregulated *KiSS-1* and *GPR54* expression in Leydig cells. Interestingly, a higher concentration of 100 μM led to reduced testosterone levels in prepubertal Shiba goats, suggesting a dose-dependent biphasic effect [103].

In females, kisspeptin neurons cooperate with NKB to initiate puberty through interneuronal communication, with both peptides co-localized in the same hypothalamic neurons [5, 63]. Similar to male models, the absence of kisspeptin or NKB results in failure of pubertal development in female sheep [3]. In ewes approaching puberty, numerous kisspeptin-immunoreactive fibers form close appositions with GnRH neurons, and this connectivity increases significantly following puberty. In addition, kisspeptin expression in the ARC of the hypothalamus rises during the normal reproductive cycle, underscoring its importance in the maintenance of female reproductive function [63].

### Kisspeptin as a potent stimulator of GnRH secretion

Kisspeptin has been widely recognized as one of the most potent stimulators of GnRH secretion in adult animals. Sharma *et al*. [104] have demonstrated that exogenous administration of kisspeptin effectively stimulates the release of GnRH, LH, and FSH from the pituitary gland. In female ruminants, two distinct modes of GnRH secretion have been characterized: A pulsatile mode and a surge mode. Pulsatile GnRH secretion governs the baseline release of LH and FSH, essential for follicular development and steroidogenesis. In contrast, the surge mode, typically occurring during the preovulatory phase of the estrous cycle, is responsible for inducing the LH surge that triggers ovulation and corpus luteum formation.

Pulsatile GnRH secretion is tightly regulated through a negative feedback loop involving sex steroids secreted by developing follicles and the corpus luteum. On the other hand, surge-mode secretion is governed by a positive feedback mechanism, wherein elevated estradiol levels from mature follicles stimulate a massive release of GnRH, ultimately leading to ovulation [66].

Recent evidence suggests that ARC kisspeptin neurons – particularly those co-expressing NKB and dynorphin (collectively termed KNDy neurons) – are critical components of the GnRH pulse generator. Within this neuronal network, NKB exerts a stimulatory effect while dynorphin provides inhibitory input, together regulating kisspeptin activity. This interplay drives the rhythmic oscillations in kisspeptin output that translate into pulsatile GnRH release [105]. Moreover, kisspeptin has been hypothesized to modulate irregular GnRH firing patterns during specific reproductive states. The majority of hypothalamic GnRH neurons in rodents express the KiSS-1R, and *ex vivo* studies using rat hypothalamic explants have confirmed kisspeptin’s stimulatory action on GnRH release. Additionally, *in vitro* experiments have demonstrated that kisspeptin can pharmacologically stimulate gonadotropin production in pituitary cells and tissue explants [43].

### Sex steroid-mediated feedback regulation of GnRH secretion

Sex steroids, particularly estrogen and progesterone, are principal modulators of the gonadotropic axis and exert dual regulatory control, positive and negative, over GnRH secretion. Estrogen, for example, can elicit a preovulatory LH surge in females during the follicular phase, representing a classic example of positive feedback. However, this effect is limited to specific stages of the ovarian cycle and is not observed under basal conditions.

In ewes, *KiSS-1* mRNA expression in the ARC increases significantly during the late follicular phase of the estrous cycle, when circulating estrogen levels are at their peak. During the lambing phase – characterized by heightened estrogenic activity – kisspeptin neurons in the ARC receive enhanced synaptic input compared to those in the luteal phase. This dynamic highlights the involvement of ARC kisspeptin neurons in mediating estrogen-induced negative feedback on GnRH secretion.

In contrast, kisspeptin neurons located in the POA and AVPV are implicated in positive feedback regulation. Estrogen acts directly on these neurons to enhance kisspeptin expression, facilitating the preovulatory GnRH surge [57]. The dichotomous role of kisspeptin neurons, negative feedback in the ARC and positive feedback in the POA/AVPV, is essential for coordinating reproductive hormone dynamics across the estrous cycle.

**Figure 6:**
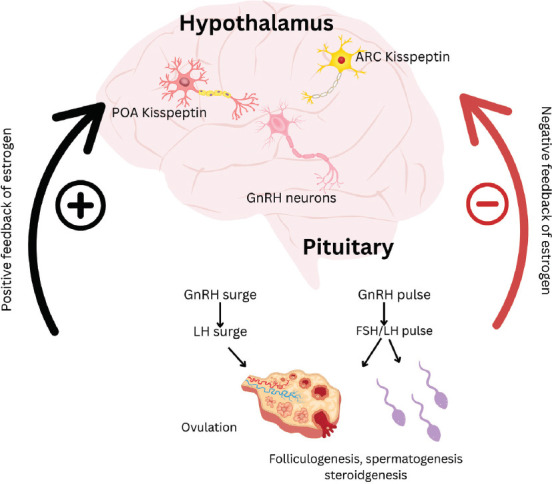
The mechanism of kisspeptin-mediated positive and negative feedback control of gonadotropin-releasing hormone secretion by sex steroids [106].

The dual action of kisspeptin in integrating sex steroid feedback is illustrated in Figure 6 [106], which depicts how estrogen modulates GnRH release through region-specific activation or suppression of kisspeptin neurons.

## LEP ACTIONS DURING SMALL RUMINANT REPRODUCTION

LEP influences reproductive physiology in small ruminants through both central and peripheral mechanisms. It modulates the HPG axis and exerts direct effects on the ovaries, uterus, oocytes, and developing embryos. Genetic polymorphisms in the *LEP* gene (*LEP/Sau3AI*) and its receptor (*LEPR/T945M*) have been linked to economically significant reproductive traits, including milk yield, lambing interval, and age at first lambing [107]. Fertile animals have been shown to exhibit higher circulating LEP levels than repeat breeders, suggesting LEP’s role in reproductive efficiency [108]. Ninpetch *et al*. [108] have confirmed a relationship between *LEP* gene polymorphisms, circulating LEP concentrations, and reproductive outcomes in small ruminants.

### Central effects of LEP on the HPG axis

LEP plays a crucial role in regulating energy homeostasis, feed intake, and neuroendocrine signaling. Its central reproductive action is primarily mediated through the HPG axis. The hypothalamus, the key integrative center for metabolic and endocrine cues, expresses LEP receptors (ObRs), particularly in the ARC and ventromedial hypothalamus, as well as in the anterior pituitary of several species, including pigs, ewes, rats, and mice [109, 110].

The LEP receptor gene (*obR*) encodes a cytokine receptor with six isoforms generated through alternative splicing of 20 exons. These include four short forms (*obRa*, *obRc*, *obRd*, and *obRf*), a long form (*obRb*), and a soluble form (*obRe*) [111]. The long isoform *obRb* (1,162 amino acids) mediates the biological functions of LEP through its interaction with Janus kinase-2 (JAK2), a cytoplasmic tyrosine kinase. Binding of LEP to *obRb* activates JAK2 autophosphorylation and downstream intracellular signaling cascades [112].

LEP has been shown to increase the pulsatility of GnRH in the ARC without affecting pulse amplitude, indicating a modulatory role rather than a direct initiator [113]. GnRH neurons – key regulators of the HPG axis – are primarily modulated by neurotransmitters such as GABA, as well as neuropeptides including kisspeptin and NPY. Although LEP does not act directly on GnRH neurons (which lack LEPRs), it exerts its influence through intermediary pathways involving kisspeptin neurons [114].

In fasted ewes, LEP administration restores LH levels, highlighting its role as a permissive factor for the onset of puberty [115]. However, LEP alone does not stimulate LH secretion in prepubertal ewes, as shown in a study where 10-day LEP treatment failed to induce LH pulses [28]. Similar findings in goats demonstrate that LEP administration affects LH secretion only under specific metabolic conditions, such as in fasted or energy-flushed animals, but not in those fed ad libitum [116].

LEP concentrations typically rise from the preovulatory to the follicular phase, implicating LEP in the LH surge and ovulatory mechanisms. Administration of LEP at physiological concentrations (10^-8^–10^-7^ M) enhances FSH secretion, whereas supraphysiological doses (>10^-5^ M) reduce FSH levels, and sub-threshold levels (<10^-8^ M) exert no measurable effect [117].

### Sexual dimorphism and nutritional influence on LEP levels

Sexual dimorphism in LEP concentrations has been documented in sheep, with rams consistently exhibiting lower levels than females, consistent with findings in other mammalian species. Prepubertal ewe lambs display significantly higher plasma LEP levels than male lambs of comparable age and nutritional status [118]. Similarly, adult ewe lambs have been reported to exhibit greater LEP concentrations than both intact and castrated rams [119]. These differences, while genetically influenced, are also modulated by environmental factors, such as dietary intake and body fat composition. Obesity and overnutrition can elevate LEP levels, although this may lead to LEP resistance, which negatively impacts reproductive function.

#### Peripheral effects of LEP on the ovary and uterus

Ovarian follicular development is regulated by intricate interactions between gonadotropins, local growth factors, and metabolic signals. LEP has been shown to influence folliculogenesis by modulating granulosa cell proliferation, steroid hormone production, and apoptotic pathways [120]. *In vitro* studies using ovarian cells from various species (rats, cattle, pigs, and humans) have demonstrated that LEP can either stimulate or inhibit the secretion of key reproductive hormones such as progesterone, androgens, and estradiol. In goats, fasting-induced reductions in circulating LEP have been linked to impaired luteal function and disruptions in estrus, suggesting LEP’s involvement in ovarian regulation under metabolic stress [121].

LEP exerts both inhibitory and stimulatory actions on ovarian function:

- Inhibitory actions:
  1. LEP suppresses insulin, IGF-1, transforming growth factor-β, and glucocorticoid-induced steroidogenesis in granulosa cells.
  2. Acute LEP administration in gonadotropin-primed immature animals has been shown to inhibit ovulation.
  3. In preantral follicles, LEP interferes with FSH-induced growth and maturation [122].

- Stimulatory actions:
  1. LEP accelerates the onset of puberty in small ruminants [123].
  2. It promotes ovulation in the absence of GnRH, especially in animals pre-treated with equine or human chorionic gonadotropins.
  3. LEP activates intracellular signaling pathways, namely JAK2/STAT3 and MEK1/2, to enhance oocyte meiotic maturation and developmental competence in rabbit models [124].

#### Direct effects of LEP on oocytes and embryos

An *in vitro* study by Alshaheen *et al*. [125] demonstrated that LEP supplementation at concentrations of 10, 100, and 1000 ng/mL in culture media accelerates preimplantation embryo development and increases blastocyst cell numbers, particularly in the trophectoderm layer. LEP modulates oocyte maturation through the activation of specific transcription factors, particularly via the STAT3 pathway, which is crucial for nuclear maturation. The surrounding cumulus cells significantly enhance LEP signaling by mediating LEP–STAT3 communication during oocyte meiosis. Notably, only the full-length isoform of the LEPRb contains the intracellular domains required to activate the JAK2/STAT3 and MAPK pathways, which are essential for these reproductive functions [126].

#### Correlation between BCS and reproductive performance in small ruminants

BCS serves as a reliable indicator of nutritional status, energy balance, and reproductive potential in goats and sheep [127]. Regular monitoring of BCS – especially during critical periods such as pre-drying, lambing, post-lambing (30–60 days), mating preparation, and breeding – can optimize reproductive outcomes. BCS assessments typically involve palpating skeletal landmarks such as the hooks, pins, transverse and spinous processes, tailhead, and rib region [128].

BCS is directly correlated with internal energy reserves and has been linked to reproductive traits including estrus onset, ovulation rate, fertility, and gestational success. It influences hypothalamic GnRH activity and pituitary sensitivity to GnRH and indirectly modulates ovarian hormone profiles and the neuroendocrine responsiveness of the HPG axis [37].

The recommended BCS range for dairy goats during the rearing period is 3.0–3.5 (on a 5-point scale) [128]. A minimum BCS of 2.75 is suggested to minimize the risk of seasonal anestrus. Higher BCS is associated with earlier estrus onset, regular cycles, higher service rates, and improved conception. Conversely, a low BCS is associated with ovarian dysfunction, including anovulation, and reduced levels of cholesterol, glucose, calcium, and magnesium in follicular fluid [127, 129]. Enhanced reproductive performance in high-BCS goats may be attributed to elevated insulin and glucose levels, which improve metabolic support for reproductive function [128].

BCS should ideally not exceed 3.5 to avoid excessive adiposity, which can also impair reproduction. Goats with BCS 3.5 show the lowest incidence of anestrus, whereas those with a BCS of 1.5 exhibit the highest [130]. Proper energy reserves are essential for maintaining fertility, particularly in dairy cows. Sitaresmi *et al*. [127] and Yilmaz *et al*. [131] have consistently reported that higher BCS at mating correlates with increased fecundity and larger litter sizes. Furthermore, ewes with BCS 3 during mid-pregnancy demonstrate higher maternal behavior scores compared to those with BCS 2. However, both extremely low and excessively high BCS values have been linked to dystocia, indicating the importance of maintaining optimal BCS for reproductive health [132].

#### Effects of kisspeptin excess and deficiency

Kisspeptin, a neuropeptide primarily produced in the hypothalamus, plays a crucial role in regulating reproductive function by controlling the HPG axis. It is synthesized by two major neuronal populations located in the rostral periventricular region of the third ventricle (RP3V) and the ARC. Kisspeptin’s primary role is the stimulation of GnRH neurons, which promotes the secretion of LH and FSH, critical hormones for folliculogenesis, ovulation, and reproductive cyclicity.

Energy balance has been shown to exert a modulatory effect on kisspeptin signaling. Undernutrition or overnutrition can suppress the kisspeptin system, leading to altered reproductive output. In such cases, reduced expression of *KiSS-1* and its receptor *KiSS-1R* (GPR54) contributes to attenuated LH pulsatility and disruption of HPG axis activity, particularly during periods of physiological or metabolic stress [13, 133].

Kisspeptin functions in tandem with GnIH, forming a dual regulatory system: kisspeptin stimulates, whereas GnIH suppresses, reproductive function. Their opposing actions respond dynamically to internal (e.g., hormonal status and energy reserves) and external (e.g., photoperiod and nutrition) cues, thereby fine-tuning reproductive activity in small ruminants [134].

Deficiency in kisspeptin or its receptor results in significant reproductive dysfunction. In various species, including humans, inactivating mutations in *KiSS-1R*, *TAC3* (NKB), or *TACR3* (its receptor) lead to pubertal failure and hypogonadotropic hypogonadism [135]. Experimental models demonstrate that a functional kisspeptin response is necessary to restore GnRH-induced LH pulsatility and resume normal pubertal progression [98]. Although direct evidence in goats remains limited, kisspeptin deficiency in other mammals has been associated with reduced ovulation rates and impaired oocyte quality, underscoring the need for species-specific research to improve reproductive health and productivity in goats.

#### Effects of LEP excess and deficiency

LEP, an adipocyte-derived hormone, is a critical regulator of energy balance, feed intake, immune function, and reproduction in small ruminants. In goats, LEP influences multiple physiological processes, including growth, lactation, metabolic stability, and reproductive performance. Administration of recombinant bovine LEP has been shown to enhance feed intake, body weight gain, and milk yield in lactating goats [136].

LEP also contributes to reproductive function by acting on the hypothalamus to modulate GnRH secretion and influence the production of LH and FSH. It is particularly important for signaling sufficient energy reserves necessary for the onset of puberty, estrus expression, and successful conception. Moreover, LEP has been implicated in reducing the incidence of metabolic disorders such as fatty liver, ketosis, and mastitis, thereby supporting overall health and reproductive capacity [33, 137].

However, chronic caloric overconsumption may lead to LEP excess and the development of LEP resistance, a condition in which the sensitivity of LEPRs is diminished. This results in impaired regulation of appetite and energy metabolism, often culminating in obesity. In goats, LEP resistance can adversely affect reproductive performance by disrupting hormonal signaling pathways essential for ovarian function, estrus behavior, and fertility [138].

In contrast, LEP deficiency, often associated with negative energy balance or malnutrition, may result in delayed puberty, anestrus, and reduced conception rates due to insufficient stimulation of the reproductive axis. Thus, optimal LEP concentrations are crucial for maintaining reproductive efficiency in small ruminants.

#### Kisspeptin and LEP as potential reproductive markers and therapeutic agents in small ruminants

Kisspeptin and LEP, due to their regulatory roles in reproduction and energy metabolism, present significant potential as genetic markers and therapeutic targets for improving reproductive performance in small ruminants. Their protein functions and polymorphic variants influence key reproductive traits, including litter size, puberty onset, and hormone secretion.

#### Kisspeptin: Genetic markers and therapeutic potential

Kisspeptin has been identified as a central regulator of the HPG axis. In Indian goat breeds, SNPs such as *g2540 C*>T in the *KiSS1* gene (with CT and TT genotypes predominating over CC) have been shown to have a potential association with reproductive traits [139]. In the Jining Gray goat, polymorphisms in intron 1 (*G296C, G45T*, and *T505A*) and exon 3 (*G3433A* and *C3688A*) are associated with improved fecundity. Similarly, Chinese goats exhibit SNPs (*G484G>A, G1147T>C, G1317G>A, G1428_1429delG*, and *G2124C>T*) linked to higher litter size [140]. In goats, SNPs in exons 1–3 of the *KiSS1* gene are more strongly associated with litter size than mutations in exons 4–12, likely due to their greater impact on mRNA stability and protein structure [140]. In ewes, polymorphisms in exons 1, 2, and 5 are also correlated with fecundity traits [141].

Mutations in *KiSS1* or *GPR54* impair reproductive development, delay puberty, disrupt Leydig cell activity, and alter gonadal function, confirming the gene’s critical role in reproductive maturation [98, 135]. However, further mechanistic studies are warranted to investigate how LEP resistance might influence kisspeptin signaling and its downstream effects on pubertal timing and fertility [7].

Therapeutically, kisspeptin shows promise in enhancing reproductive function. Administration of 10 μM kisspeptin for 24 h has been shown to increase testosterone production in goat Leydig cells [13]. Subcutaneous injection of kisspeptin-10 (KP-10) at doses of 5 μg/kg body weight (BW) in male Shiba goats [142] and 1.5–10 mg/kg BW in females [143] significantly stimulates GnRH and LH secretion. In addition, KP-10 injection in ovariectomized goats and ewes elevates circulating GnRH and steroid hormones such as estrogen and progesterone [143]. Kisspeptin analogs have also been reported to enhance milk production in ruminants [144], potentially through improved neuroendocrine stimulation of lactogenesis.

#### LEP: Polymorphisms and therapeutic implications

LEP contributes to reproductive physiology by modulating the metabolic-reproductive axis, including lactogenesis, litter size, nutritional balance, and HPG axis activity [33]. In goats, polymorphisms in *LEP*, particularly in intron 1, influence RNA expression, feed intake, and nutrient assimilation [145]. These variations affect metabolic hormones, thyroid function, and feeding behavior, thereby influencing reproductive efficiency [146].

In males, mutations at the *LEP* 170G>A locus have been associated with altered sperm motility and viability. Another SNP, 332G>A in the Sanjabi breed, has been linked to infertility, reflecting the deleterious effects of amino acid changes on reproductive protein function [147, 148]. In females, *LEPR* polymorphisms have been associated with seasonal estrus expression, puberty onset, gestational success, and milk yield [149, 150]. These associations may be mediated by the receptor’s expression in the suprachiasmatic nucleus – a key circadian regulator – and its communication with the pineal gland.

Therapeutically, LEP administration at 1–100 ng/mL has been shown to enhance oocyte nuclear maturation by activating the MAPK and JAK2/STAT3 signaling pathways [120]. LEP injections at various physiological doses stimulate the onset of estrus in seasonally breeding ewes and does [115], and infusion of 1–25 μg/h for 8 days has been reported to elevate GH, FSH, and LH pulse frequency in ewes [151].

#### Marker-assisted selection and therapeutic applications

The genetic variability of *KiSS1*, *LEP*, and their respective receptors (*GPR54* and *LEPR*) holds significant promise for marker-assisted selection programs aimed at enhancing reproductive traits in goats and sheep. In addition, their physiological functions position them as candidates for therapeutic intervention in managing infertility, delayed puberty, and other reproductive disorders. The strategic use of kisspeptin and LEP analogs or agonists could help sustain reproductive performance in metabolically challenged or subfertile livestock, offering both genetic and pharmacological solutions for enhancing productivity in small ruminant systems.

## CONCLUSION

This review consolidates the pivotal roles of kisspeptin and LEP in regulating reproductive physiology in small ruminants, emphasizing their mechanistic influence on the HPG axis, metabolic integration, and reproductive hormone secretion. Kisspeptin acts as a central stimulator of GnRH, LH, and FSH release, initiating and maintaining reproductive cyclicity, pubertal onset, and ovulation. LEP, a metabolic hormone, complements kisspeptin signaling by conveying energy availability to the reproductive axis and modulating the neuroendocrine response to nutritional status.

Practical applications of these findings include the use of *KiSS1* and *LEP* gene polymorphisms as candidate markers in genetic selection programs aimed at improving reproductive traits such as litter size, puberty onset, and fertility rates. Moreover, the administration of kisspeptin and LEP analogs offers therapeutic potential for overcoming reproductive inefficiencies, especially under metabolic or seasonal constraints. Controlled kisspeptin or LEP dosing has been demonstrated to stimulate ovulation, support oocyte maturation, and enhance steroidogenesis, even in subfertile or prepubertal animals.

The strengths of this review lie in its integrative focus, encompassing molecular, physiological, and applied aspects of reproductive endocrinology in small ruminants. It provides comparative insights into gene polymorphisms across breeds and highlights synergistic interactions between metabolic and reproductive signaling pathways.

However, certain limitations exist. Most mechanistic data on kisspeptin and LEP originate from rodent or human models, with limited functional validation specific to goats and sheep. In addition, while several polymorphisms have been statistically associated with reproductive traits, functional causality and tissue-specific expression studies remain sparse. The interactions between LEP resistance and kisspeptin signaling under field conditions also remain poorly understood.

Future research should prioritize functional genomics to validate causal SNPs, explore LEP–kisspeptin cross-talk in energy-deficient states, and assess the long-term impacts of exogenous hormone administration on fertility, offspring viability, and endocrine homeostasis. Advancing transcriptomic and proteomic profiling in hypothalamic and gonadal tissues across physiological stages will provide deeper insights into the regulation of reproductive competence.

Kisspeptin and LEP emerge as promising reproductive biomarkers and therapeutic agents in small ruminant production systems. Their strategic integration into breeding and reproductive management programs could enhance fertility outcomes, especially in nutritionally challenged environments. Unlocking their full potential will require translational research linking molecular insights to field-based applications in goat and sheep husbandry.

## AUTHORS’ CONTRIBUTIONS

HH, II, PIS, and DAM: Conceived and designed the study. SS, PIS, MFH, and DAM: Performed the experiments and collected the data. MAC, FBIL, NA, AH, RIA, SYH, and PIS: Data analysis and interpretation. PIS, SS, HH, and DAM: Drafted the manuscript. PIS, II, SS, and HH: Coordinated project administration and supervised the research activities. All authors have read, reviewed, and approved the final manuscript.
